# Long-Range Temporal Correlations in Alpha Oscillations Stabilize Perception of Ambiguous Visual Stimuli

**DOI:** 10.3389/fnhum.2018.00159

**Published:** 2018-04-24

**Authors:** Francesca Sangiuliano Intra, Arthur-Ervin Avramiea, Mona Irrmischer, Simon-Shlomo Poil, Huibert D. Mansvelder, Klaus Linkenkaer-Hansen

**Affiliations:** ^1^IRCCS, Don Gnocchi Foundation, Milan, Italy; ^2^Department of Integrative Neurophysiology, CNCR, Amsterdam Neuroscience, Vrije Universiteit Amsterdam, Amsterdam, Netherlands; ^3^NBT Analytics BV, Amsterdam, Netherlands

**Keywords:** bistable perception, spontaneous brain fluctuations, voluntary control, resting-state EEG, resting-state questionnaire

## Abstract

Ongoing brain dynamics have been proposed as a type of “neuronal noise” that can trigger perceptual switches when viewing an ambiguous, bistable stimulus. However, no prior study has directly quantified how such neuronal noise relates to the rate of percept reversals. Specifically, it has remained unknown whether individual differences in complexity of resting-state oscillations—as reflected in long-range temporal correlations (LRTC)—are associated with perceptual stability. We hypothesized that participants with stronger resting-state LRTC in the alpha band experience more stable percepts, and thereby fewer perceptual switches. Furthermore, we expected that participants who report less discontinuous thoughts during rest, experience less switches. To test this, we recorded electroencephalography (EEG) in 65 healthy volunteers during 5 min Eyes-Closed Rest (ECR), after which they filled in the Amsterdam Resting-State Questionnaire (ARSQ). This was followed by three conditions where participants attended an ambiguous structure-from-motion stimulus—Neutral (passively observe the stimulus), Hold (the percept for as long as possible), and Switch (as often as possible). LRTC of resting-state alpha oscillations predicted the number of switches only in the Hold condition, with stronger LRTC associated with less switches. Contrary to our expectations, there was no association between resting-state Discontinuity of Mind and percept stability. Participants were capable of controlling switching according to task goals, and this was accompanied by increased alpha power during Hold and decreased power during Switch. Fewer switches were associated with stronger task-related alpha LRTC in all conditions. Together, our data suggest that bistable visual perception is to some extent under voluntary control and influenced by LRTC of alpha oscillations.

## Introduction

**Figure 1:**
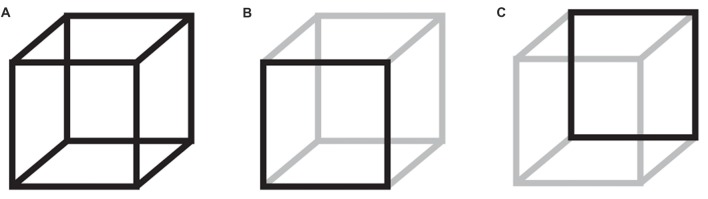
The Necker cube bistable illusion. When participants attend a 3-dimensional cube, **(A)** they perceive, alternately, the bottom left **(B)** or the top right **(C)** as the front of the cube. The neural mechanisms that govern these perceptual switches are not entirely understood.

The constructive nature of perception becomes apparent in multistable perception, where an unchanging physical stimulus lends itself to multiple, mutually exclusive interpretations. One such example is the Necker cube (Necker, 1832), where either the bottom left or the top right square can be perceived, alternately, as the front or back of a 3-dimensional cube (Figure 1). In lack of any additional depth cues, the conscious percept will switch between the two alternatives. Top-down processes have been implicated with the initiation of new percepts; however, the spontaneous and stochastic nature of percept switching points to a role also of noisy brain fluctuations (Gigante et al., 2009; van Ee, 2009; Huguet et al., 2014; Meso et al., 2016).

The notion that neuronal noise influences perception is not a new concept, e.g., fluctuations in spontaneous activity as measured with functional magnetic resonance imaging have been related to inter-trial variability in perception (Fox et al., 2006). What aspects of brain fluctuations influence multistable perception? Jensen and Mazaheri (2010) have proposed that alpha oscillations regulate neuronal network excitability through a mechanism of functional inhibition. In this framework, lower alpha power should lead to excitability windows with a low threshold for perceptual discrimination—possibly increasing the probability of perceptual switches—whereas higher alpha power should increase the discrimination threshold and promote percept maintenance (Lange et al., 2014). This idea is supported by the finding that a reduction in alpha power precedes perceptual switches (Isoglu-Alkaç et al., 2000; Strüber and Herrmann, 2002; Isoglu-Alkaç and Strüber, 2006; Piantoni et al., 2017), and that the extent to which alpha power increases right after a perceptual switch predicts how long the percept stays stable (Piantoni et al., 2017). Interestingly, patients with schizophrenia have reduced alpha power during rest (Sponheim et al., 2000) and are less capable of maintaining percepts stable for a long time (McBain et al., 2011), which is in line with a relationship between alpha power and percept maintenance (Piantoni et al., 2017).

The delta and gamma bands have also been associated with perceptual reversals of bistable stimuli, with increases in delta (Müller et al., 2005; Mathes et al., 2006; Ozaki et al., 2012) and gamma power occurring right before or during the perceptual changes (Başar-Eroglu et al., 1996; Strüber et al., 2000, 2001; Mathes et al., 2006). These have been interpreted as top-down signals that trigger perceptual reversals, evidence for causality coming from studies that used transcranial alternating current stimulation at gamma frequency over the posterior cortex, which led to an increased number of perceptual switches (Strüber et al., 2014; Cabral-Calderin et al., 2015).

While momentary brain fluctuations influence perception of ambiguous stimuli over the course of time within each individual, some aspects of bistable perception, such as the distribution of percept durations, are characteristic for each individual. To explain the lack of short percepts in some participants, Kloosterman (2015) proposed a stabilizing mechanism comprised of low-level neuronal adaptation and top-down signals, which acts on the percept after a reversal, and varies in strength across individuals. On a related note, Hancock (2013) suggested that genetic factors related to sinistrality impact the level of neuronal noise responsible for spontaneous switching and, subsequently, affect the percept dominance distributions. Furthermore, Dowlati et al. (2016) showed that perceptual beliefs are more likely to bias bistable perception in older individuals, possibly owing to a heightened role of top-down processes.

Here, we investigate whether resting-state activity, which shows stable individual differences in oscillation power (Smit et al., 2005) and long-range temporal correlations (LRTC; Linkenkaer-Hansen et al., 2007), could explain inter-individual differences in the anatomical and functional organization of the underlying networks with implications for perception. We consider alpha oscillations as a source of neuronal noise impacting bistable perception, possibly through a mechanism of gating via functional inhibition or top-down control for which the role of LRTC is poorly understood. Therefore, we define two aspects of this neuronal noise: the level of noise, as reflected in the power of alpha oscillations, and the temporal structure of noise, reflected in the strength of LRTC.

When participants had to tap their finger rhythmically every 1 s without feedback, the temporal structure of alpha oscillations during resting-state predicted the temporal structure of timing errors in a subsequent finger-tapping task (Smit et al., 2013). In another study, the temporal structure of the distribution of hits and misses in auditory and visual threshold detection tasks has been associated with individual differences in LRTC of neuronal oscillations (Palva et al., 2013). We hypothesize that the temporal structure of alpha oscillations in the resting-state is also predictive of behavior in bistable perception. We consider that a more chaotic state, characterized by reduced temporal correlations, will lead more often to percept destabilization. Conversely, we expect that strengthening the LRTC of alpha oscillations should have the effect of stabilizing the percepts for longer durations, leading to less spontaneous switches.

It is also known that participants differ in the content of thoughts experienced during resting-state (Hurlburt et al., 2015); however, the type of mentation occurring during rest is relatively stable within individuals (Stoffers et al., 2015). Thus, it is plausible that individual differences in thoughts during rest are predictive of attention and perception performance. In particular, we hypothesized that Discontinuity of Mind—as measured by the Amsterdam Resting-State Questionnaire (ARSQ; Diaz et al., 2013, 2014)—reflects a more noisy organization of neuronal and cognitive systems. Therefore, individuals with greater continuity of thoughts should experience less spontaneous switches during viewing of a bistable visual stimulus.

## Materials and Methods

### Eyes-Closed Rest (ECR)

The classical Eyes-Closed Rest (ECR) recording of continuous electroencephalography (EEG) was performed for 5 min while participants were seated with eyes closed. The instruction was: “Please sit in a comfortable position, relax, keep your eyes closed, move as little as possible and try not to fall asleep.” After 5 min a tone signaled them to open their eyes and to fill in the ARSQ.

### Amsterdam Resting-State Questionnaire (ARSQ)

To investigate thoughts and feelings experienced during rest participants completed the ARSQ following the ECR recording. The ARSQ consists of 55 statements presented on the computer screen and rated on a 5-points Likert scale from completely disagree to completely agree (Diaz et al., 2014). The statements are grouped into 10 factors labeled: Discontinuity of Mind, Theory of Mind, Self, Planning, Sleepiness, Comfort, Somatic Awareness, Health Concern, Visual Thought and Verbal Thought.

### Structure From Motion (SFM)

**Figure 2:**
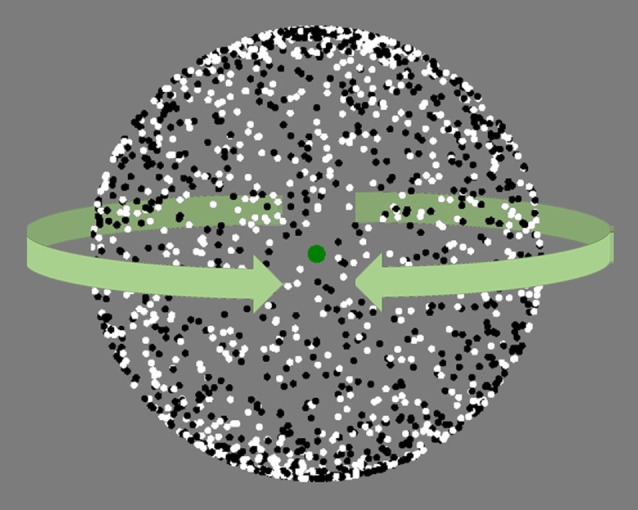
Snapshot of the structure from motion (SFM) stimulus. The white and black dots move continuously from left to right and, once they reached the edge, from the right to the left. The dots moving from left to right tend to be perceived as the back or front of the sphere, creating the 3D illusion of a ball turning clockwise or counter clockwise. SFM stimulus same as in van Loon et al. (2013).

In the next step, we investigated perceptual switches with the Structure From Motion (SFM) task (van Loon et al., 2013), while EEG was recorded. The SFM is a bistable stimulus in which a cloud of moving dots can give rise to the perception of a sphere moving clockwise or counterclockwise (Figure 2). The perceived rotation of the sphere spontaneously changes direction at irregular intervals. The SFM stimulus was presented for 133 s in three blocks. The task instruction differed for every block and, therefore, each block was preceded by a 20-s training period. In the first block, named Neutral, participants were instructed to passively observe the stimulus. For the second block, Switch, they were asked to observe the same stimulus and attempt to flip the percept as often as possible. For the third block, Hold, they were asked to maintain the percept as stable as possible, i.e., try to maintain the perceived direction of motion at any given moment. To prevent eye movements from destabilizing the percepts and initiate reversals, we required participants to fixate a green dot at the center of the screen. For all three blocks participants were required to report switches by pressing the Z (for the leftward turning percept) and X (for the rightward turning percept) keys on the keyboard. After each block, participants rated their Feeling of Control of the perceptual switches using a Likert scale from 1 to 7 where 1 corresponds to “completely uncontrolled” and 7 corresponds to “completely controlled”.

### Data Acquisition

Recordings were performed using an Electrical Geodesics (EGI, Eugene, OR, USA) EEG system (GES400 DC), with 128-EGI HydroCel channel sponge-based EEG caps. Impedances were kept below 70 kOhm (a suitable threshold for this EEG system). Data were recorded at 1000 Hz sampling rate, with a 400 Hz low-pass (GES250), 0.01 Hz high-pass hardware filter and with 16 Bit resolution and reference at Cz (vertex).

### Participants

The 65 participants were healthy students of the Vrije Universiteit Amsterdam, as well as healthy volunteers from the general population, overall ranging between 20 years and 48 years (mean = 25 years, *SD* = 6.2; 35 females). This study was carried out in accordance with the recommendations of the ethics committee of the Vrije Universiteit Amsterdam, with written informed consent from all subjects. All subjects gave written informed consent in accordace with the Declaration of Helsinki. The protocol was approved by the ethics committee of the Vrije Universiteit Amsterdam. The tasks were developed in OpenSesame and were displayed on a 60 Hz, Dell E178FP monitor with a resolution of 1280 × 1024 pixels, viewed at a distance of 50 cm. Participants underwent the SFM tasks as described above. For 55 of the participants, we also recorded, right before the SFM, 5 min of ECR, after which they filled in the ARSQ describing the content of their thoughts throughout the ECR.

### EEG and ARSQ Data Analysis

The statistical analysis of EEG and ARSQ data was performed using the Neurophysiological Biomarker Toolbox (Hardstone et al., 2012), available at www.nbtwiki.net. First, noisy channels were removed, then, remaining signals were inspected in windows of 10 s, and transient artifacts, for example, caused by head movements were manually marked and omitted from the subsequent analyses. The average duration of the cleaned signals was 291 s (ranging from 255 s to 300 s) for the 300 s ECR recordings, and 125 s (ranging from 85 s to 133 s) for the 133 s SFM recordings. We used Independent Component Analysis (Bell and Sejnowski, 1995) to identify eye-movement artifacts and project these out of the EEG data. Subsequently, quantitative measures of oscillatory activity were computed. We refer to these values as biomarkers. Further, we removed five participants due to extreme values in the alpha and delta absolute power (deviating more than six standard deviations from the mean), thus leaving a total of 60 participants for the SFM tasks, and 50 participants for ECR.

The Alpha band was defined as 8–13 Hz. Absolute power was estimated by applying Welch’s modified periodogram method implemented in Matlab as *pwelch()*, with non-overlapping Hamming windows of 1 s followed by computing the square root of the power spectrum obtained. The Detrended Fluctuation Analysis (DFA) was used to analyze the scale-free decay of temporal (auto)correlations in the amplitude envelope of neuronal oscillations, also known as LRTC. The DFA was introduced as a method to quantify correlations in complex data with less strict assumptions about the stationarity of the signal than the classical autocorrelation function or power spectral density (Linkenkaer-Hansen et al., 2001; Hardstone et al., 2012). An additional advantage of DFA is the greater accuracy in the estimates of correlations, which facilitates a reliable analysis of LRTC up to time scales of at least 10% of the duration of the signal (Chen et al., 2002; Gao et al., 2006). Furthermore, estimates of LRTC by means of DFA are robust to the removal of portions of the signal, even when up to 50% of the signal is cut out (Chen et al., 2002). As such, the artifact removal procedure described above should not have any discernible effects on the results. DFA exponents in the interval of 0.5–1.0 indicate scale-free temporal correlations (autocorrelations), whereas an exponent of 0.5 characterizes an uncorrelated signal. The main steps from the broadband signal to the quantification of LRTC using DFA have been explained in detail previously (Linkenkaer-Hansen et al., 2001; Hardstone et al., 2012). In brief, the DFA measures the power-law scaling of the root-mean-square fluctuation of the integrated and linearly detrended signals, *F*(*t*), as a function of time window size, *t* (with an overlap of 50% between windows). The DFA exponent is the slope of the fluctuation function *F*(*t*) and can be related to the power-law scaling exponent of the autocorrelation function. The signal was first band-passed in the alpha range using finite impulse response filters with a Hamming window of 1 s and a filter order of 250. The amplitude envelope was extracted using the Hilbert transform. *F*(*t*) was computed on time windows situated on a log-scale between 0.5 s and 80 s, with an overlap of 50%. The DFA exponent was fit between 1 s and 20 s.

### Statistical Testing

Given that the distribution of the number of switches is non-normal, and the self-rated Feeling of Control is ordinal with a restricted range, we used non-parametric statistical tests for hypothesis testing involving this data. More specifically, we used Wilcoxon’s sign-ranked test for comparing across conditions, and Spearman’s rank correlation coefficient to assess the relationship between variables. When plotting linear correlations, we showed Spearman’s *r*_s_ coefficient computed on the ranked-data, but displayed the raw values, with a least-squares line fit to the raw data, for visual guidance. Statistical comparisons between EEG biomarkers were made using the paired *t*-test. To evaluate the overall correlation between alpha-band LRTC and the number of switches, aggregated across all three SFM conditions, we used the repeated measures correlation (Bland and Altman, 1995a,b; Bakdash and Marusich, 2017). For all statistical tests, we set a *p*-value significance threshold of 0.05. To correct for multiple comparisons across the 128 electrodes, we used the binomial correction (Montez et al., 2009; Nikulin et al., 2012; Schiavone et al., 2014). According to the binomial distribution, the likelihood of having 12 significant electrodes out of 128 is <2%. As such, for every statistical test involving all 128 electrodes, we consider the effects to be significant, only when the number of significant electrodes is higher or equal to 12.

## Results

Because our primary goal was to understand how inter-individual variability during the resting state relates to variability in bistable perception, we recorded EEG during 5 min of ECR, and then while participants watched a SFM bistable stimulus (Figure 2). The SFM task consisted of three conditions: Neutral (simply watch the stimulus), Switch (the percept as often as possible) and Hold (maintain the percept for as long as possible).

### Perceptual Switches Are Under Voluntary Control

**Figure 3:**
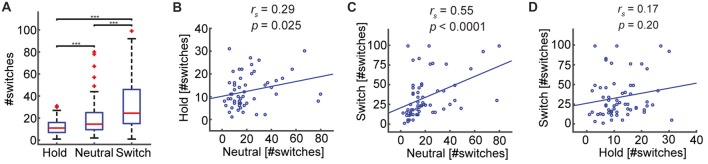
The number of perceptual switches increases or decreases according to task requirements.** (A)** Subjects can voluntarily affect percept reversals. The number of switches differs significantly across conditions (***p ≤ 0.0001), in line with the task demands. **(B,C)** Significant positive correlations indicate that participants who experience many switches during the passive viewing condition (Neutral) also experience many switches when asked to maintain the percepts stable (Hold), or when required to switch as often as possible (Switch). **(D)** The number of switches in the Hold and Switch conditions were not correlated.

To investigate whether the participants were able to voluntarily control their percepts according to the task requirements, we compared the number of switches across the three blocks: Hold (*M* = 12.28, *SD* = 7.19), Neutral (*M* = 20.32, *SD* = 17.49), and Switch (*M* = 34.05, *SD* = 26.51; Figure 3A). Participants were able to reduce the number of switches relative to the passive viewing condition, when instructed to maintain their percepts for as long as possible: Hold—Neutral (*Z* = −3.38, *p* = 0.0007; Wilcoxon’s non-parametric sign-rank test), and, conversely, increase the number of switches, when instructed to switch as often as possible: Switch—Neutral (*Z* = 4.35, *p* < 0.0001). After confirming that participants were able to exert control over the SFM percepts, we evaluated whether the rate of spontaneous switches during passive viewing, related to the rate of switching during voluntary control. Using Spearman’s rank-correlation test, we found significant correlations between the number of switches in Neutral and Hold (*ρ*_58_ = 0.29, *p* = 0.025, Figure 3B), as well as between Neutral and Switch (*ρ*_58_ = 0.55, *p* < 0.0001, Figure 3C). Thus, participants who experience more spontaneous switches during passive viewing, tend to experience more switches when attempting to control the percepts, regardless of their intention. The number of switches between the two voluntary conditions, Hold and Switch, were not correlated (*ρ*_58_ = 0.17, *p* = 0.20, Figure 3D).

### Participants Feel Partially in Control of Perceptual Switches

**Figure 4:**
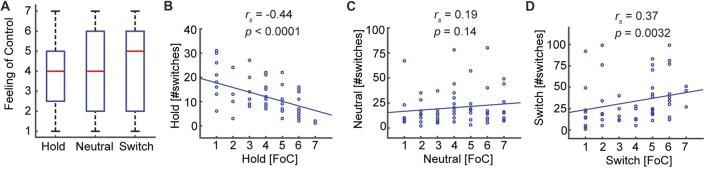
The Feeling of Control reflects subjects’ ability to conform to task requirements. **(A)** The Feeling of Control does not change between the three conditions. **(B,D)** The absolute task performance correlates with the self-reported Feeling of Control. Thus, participants reporting a high Feeling of Control experience a low number of perceptual switches in the Hold condition and a high number of switches in the Switch condition. **(C)** In the Neutral condition, where the subjects do not receive any instructions about the number of switches they should make, the Feeling of Control does not correlate with the number of switches.

That participants, on average, are able to influence the number of perceived switches does not necessarily mean that they feel in control of this process. To investigate this, we compared the self-reported Feeling of Control. Interestingly, participants reported an intermediate and equal level of control in all three conditions: Hold (*M* = 3.83, *SD* = 1.77), Neutral (*M* = 4.05, *SD* = 1.97), Switch (*M* = 4.10, *SD* = 1.94), Hold—Neutral (*Z* = −0.71, *p* = 0.48), Neutral—Switch (*Z* = −0.15, *p* = 0.88), Hold—Switch (*Z* = −1.10, *p* = 0.26; Figure 4A). There was only a trend towards the Feeling of Control correlating with the change in the number of switches relative to the Neutral condition: Hold (*r*_s 58_ = −0.245, *p* = 0.059), Switch (*r*_s 58_ = 0.252, *p* = 0.052). The Feeling of Control, however, did correlate with the absolute number of switches for the conditions Hold (*r*_s 58_ = −0.44, *p* = 0.0005, Figure 4B) and Switch (*r*_s 58_ = 0.37, *p* = 0.0032, Figure 4D), but not in the Neutral condition (*r*_s 58_ = 0.19, *p* = 0.14, Figure 4C). Thus, the Feeling of Control corresponds to a great extent to how well the participants can control their percepts, when they have an explicit intention to do so.

### Eyes-Closed Rest Oscillations Predict the Number of Switches During Hold

**Figure 5:**
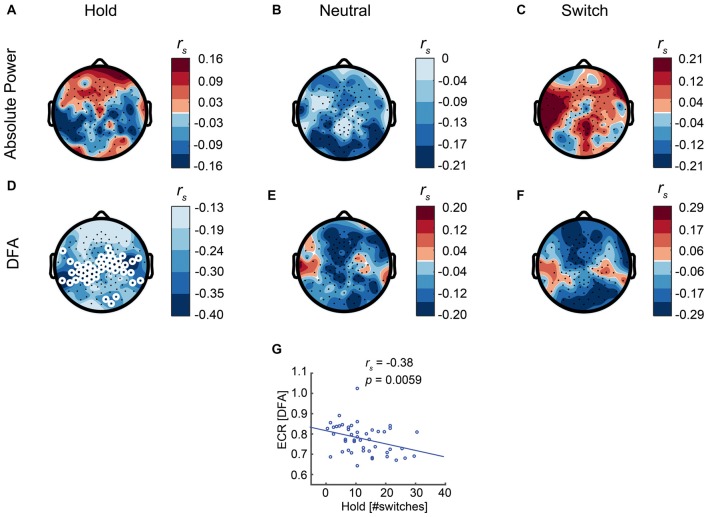
Eyes-closed rest (ECR) activity predicts the number of switches during the Hold condition. **(D)** Long-range temporal correlations (LRTC) of ECR alpha oscillations show a significant correlation with the number of switches in the Hold condition. Thereby, subjects with stronger resting-state alpha LRTC are better at maintaining stable percepts. **(E,F)** For the other conditions, there is no significant relationship between LRTC and the number of switches. **(A–C)** Absolute alpha power is not significantly correlated with the number of switches in any of the conditions. White circles represent significant electrodes. **(G)** Correlation between LRTC and the number of switches in the Hold condition, shown for the significant electrodes.

Next, we evaluated the link between resting-state brain dynamics and bistable perception. Our expectation was that participants with stronger LRTC in the alpha band and, hence, less random neural dynamics, would experience less switches. To evaluate LRTC in the alpha band, we used DFA (Hardstone et al., 2012), which quantifies the scaling of signal auto-correlations across multiple time windows, here spanning 1–20 s (see “Materials and Methods” section). A DFA of 0.5 corresponds to a memoryless signal, whereas a higher DFA closing towards 1 reflects stronger LRTC. We found that LRTC of alpha oscillations correlated negatively with the number of switches, in line with our hypothesis, albeit only for the Hold condition (*r*_s 48_= −0.38, *p* = 0.0059, Spearman’s rank-correlation test, calculated for the average across the significant electrodes, Figures 5D,G). No significant relationship was found between alpha LRTC and the number of switches in the other conditions (Figures 5E,F), or between absolute alpha power and the number of switches (Figures 5A–C).

### Regulating Perceptual Switches Is Associated With High Changes in Power and LRTC of Alpha Oscillations

**Figure 6:**
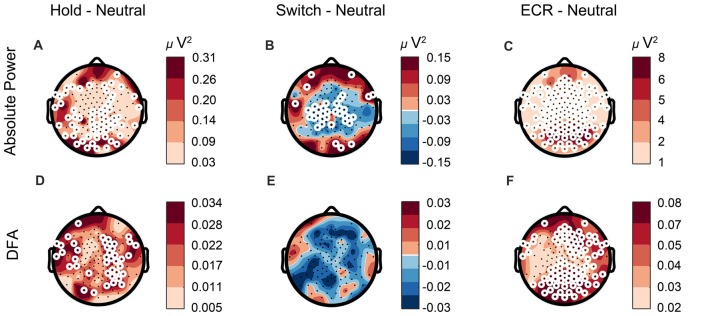
Alpha power changes according to task requirements. **(A,D)** Alpha power and LRTC are elevated in the Hold compared to the Neutral condition, whereas **(B)** alpha power is suppressed in the Switch condition. **(E)** LRTC is not significantly altered between the Switch and Neutral conditions.** (C,F)** The difference between ECR and Neutral is higher than that between any of the SFM conditions, suggesting a different cortical state during ECR compared to tasks, with increased alpha power and stronger LRTC. White circles represent significant electrodes.

Previous research has related changes in alpha power to bistable perception, such that a decrease in alpha power occurs right before a percept reversal, and the level of alpha power predicts percept duration (Piantoni et al., 2017). Since the participants were able to voluntarily reduce or increase the number of reversals, we investigated whether changes in the number of switches were reflected in alpha power measured during the task. To compare the EEG biomarkers between conditions, we used paired *t*-tests. After correcting for multiple comparisons using the binomial correction criterion, we averaged values across all significant electrodes, and compared between conditions. We found alpha power in the Hold condition to be significantly higher than the Neutral condition, in line with our expectations (*t*_59_ = 3, *p* = 0.0042, paired *t*-test, Figure 6A). For the Switch condition the pattern was more complex, showing increases in alpha at a few frontal and occipital electrodes, and widespread decreases in alpha for most central electrodes (representative central electrode, *t*_59_ = 2.76, *p* = 0.0077, Figure 6B). The LRTC tended to mirror the oscillation-power effects with an increase during Hold compared to Neutral (*t*_59_ = 4.2, *p* = 0.0001, Figure 6D), and a decrease during Switch (Figure 6E), albeit this did not reach significance. Finally, we evaluated the differences between Neutral and ECR. We found both alpha power (*t*_59_ = 8.9, *p* < 0.0001, Figure 6C) and DFA (*t*_59_ = 4.5, *p* < 0.0001, Figure 6F) to be reduced broadly across the entire scalp during passive viewing compared to ECR.

### Perceptual Instability Is Associated With Weak LRTC and—to Some Extent—Low Power of Alpha Oscillations

**Figure 7:**
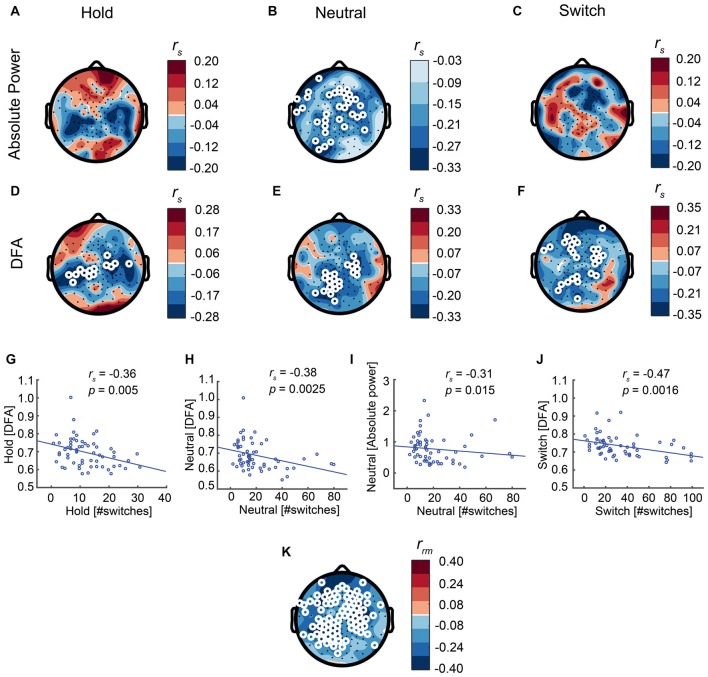
Strong LRTC is associated with fewer perceptual switches in all conditions.** (A–C)** Alpha power is associated with the number of switches only in the Neutral condition.** (D–F)** The LRTC biomarker of alpha oscillations is negatively correlated with the number of switches across each of the three conditions. White circles represent significant electrodes. **(G–J)** Correlations shown for the significant electrodes. **(K)** Inter-individual differences in alpha-band LRTC aggregated across all three SFM conditions, correlate negatively with inter-individual differences in the number of switches across the same three conditions, as calculated using repeated-measures correlation.

Our main hypothesis was that stronger resting-state LRTC in the alpha band would lead to fewer switches. Indeed, DFA correlates negatively with the number of switches across each of the SFM conditions: Hold (*r*_s 58_= −0.36, *p* = 0.005, Spearman’s rank-correlation test, calculated for the average across the significant electrodes, Figures 7D,G), Neutral (*r*_s 58_ = −0.38, *p* = 0.0025, Figures 7E,H), Switch (*r*_s 58_ = −0.47, *p* = 0.0002, Figures 7F,J), as well as when aggregated across all three conditions (repeated-measures correlation, Figure 7K). In addition, in line with previous research, we found a negative correlation between alpha power and the number of switches, albeit only during Neutral (*r*_s 58_ = −0.31, *p* = 0.015, Figures 7A–C,I), when there is no explicit instruction to control the percepts.

### Discontinuity of Mind Does Not Affect the Number of Switches

Our second hypothesis was that experiencing continuity of thoughts during rest reflects neuronal systems that are less “noisy” when compared to discontinuous thought patterns and, hence, should promote the stabilization of percepts in the visual task. Contrary to our expectation, the Discontinuity of Mind score from the ARSQ—measured right after ECR—did not correlate with the number of switches in any of the conditions: Hold (*r*_s 48_ = −0.01, *p* = 0.51), Neutral (*r*_s 48_ = −0.22, *p* = 0.12), Switch (*r*_s 48_ = −0.1, *p* = 0.47). As an exploratory test, we then investigated whether any of the other nine ARSQ dimensions would predict the number of switches in any of the three conditions; however, we did not observe any significant associations (after correcting for multiple comparisons using Bonferroni’s criterion).

## Discussion

What causes spontaneous switching during multistable perception? It is known that perceptual reversals emerge out of low-level interactions between mutually inhibiting neural populations coding for the competing percepts (Noest et al., 2007; van Loon et al., 2013), and are subject to slow adaptation dynamics (Pastukhov and Braun, 2008, 2013; Huguet et al., 2014). While this implies an influence of previous history on successive percepts, Leopold and Logothetis (1999) noticed that percept-dominance times are stochastically independent (Borsellino et al., 1972). They proposed that the function of this stochastic process is similar to that of attention sampling the environment for salient stimuli, allowing the brain to randomly explore different representations of the same stimuli. Various computational models (Moreno-Bote et al., 2007; Gigante et al., 2009; Freyer et al., 2011; Huguet et al., 2014; Meso et al., 2016), have integrated the two ideas, and suggest that while mutual inhibition and adaptation do bias the perceptual dynamics, intrinsic brain fluctuations characterized as “noise” also initiate perceptual switches. In this framework, the contribution of noise would explain the stochastic nature of spontaneous percept reversals.

### Differences in Cortical State Explain Why the Relationship of Task-Related LRTC to Percept Switching Is Stronger Than That of Resting-State LRTC

Here, we proposed that resting-state LRTC in the alpha band reflect the randomness of spontaneous brain fluctuations, and that a stronger LRTC is conducive to fewer switches, by means of a more temporally structured neuronal noise. We found a link between resting-state LRTC and behavior only in the Hold condition. On the other hand, task-related LRTC revealed this association in all conditions. Why is it that resting-state LRTC are not as powerful at predicting behavior as task-related LRTC? Clearly, task-related activity is measured at the same time with behavior, while resting-state activity is measured at an earlier time point. Still, the difference may be heightened by the ECR and SFM conditions reflecting different cortical states. Hahn et al. (2017) found that the visual cortex state in cats shifts from critical to subcritical when the animal opens their eyes. In our experiment, the weaker alpha LRTC, and reduced alpha power in Neutral when compared to ECR, is likely to reflect such a shift towards subcritical dynamics (Figures 6C,F). What may enable ECR to be predictive of behavior in Hold but not in the other two SFM conditions, is that Hold comes closest to ECR in terms of alpha power spectrum and LRTC. It is also plausible that the Hold condition invokes less top-down mechanisms and in that way resembles the resting state more.

### Relationship of LRTC With Percept-Switching Rate Not Merely a Consequence of Percept Durations

That LRTC of task-related alpha oscillations predict the number of switches seems to confirm our hypothesis that the temporal structure of neuronal noise influences bistable perception. Smaller percept durations could result in shorter time scales over which signal auto-correlations can emerge, and, in consequence, weaker LRTC. Are the stronger LRTC seen during the task simply a byproduct, rather than a cause, of a decreased number of switches? The effects that we see for task-related LRTC are similar to those observed in resting-state LRTC, even when the latter are not significant. Since ECR activity is void of task-related artifacts, this lends support to a causal relationship between LRTC and spontaneous switching. Furthermore, it is notable that the topology of resting-state and task-related LRTC effects on bistable perception corresponds to the involvement of the parietal cortex in the initiation of perceptual switches (Britz et al., 2008; Baker et al., 2015; Megumi et al., 2015).

### Importance of LRTC in Percept Stabilization

In Piantoni et al. (2017), the authors have established a causal role of alpha oscillations in perceptual switching, showing that sleep deprived participants, with increased alpha activity, experience less percept reversals. In line with these results, we found that alpha power is modulated in the SFM tasks, with low power associated with rapid percept switching and high power with percept maintenance. On the other hand, Mathes et al. (2006), studying voluntary control of the Necker cube illusion, noticed that top-down control modulates delta and gamma-band, but not alpha. It is important to note at this point that the two tasks differ. In the Necker cube task, a simple shift of attention between the front and the back of the cube can switch the percept. The SFM illusion, however, requires complex integration of motion information to form a stable percept, which makes perceptual switching more difficult to control. Helfrich et al. (2016), propose that alpha oscillations play a role in inter-hemispheric integration of motion information, which would explain why, in our experiment, the modulation of alpha power across the three SFM conditions is effective. This could relate to the role of LRTC in decreasing the number of switches. In networks with strong LRTC, external input is more likely to have lasting effects on network activity than in a network with more chaotic dynamics. As such, we speculate that connected local cortical patches with strong LRTC are more likely to entrain each other and establish synchrony. If efficient information integration across brain areas requires coherence of local network signals (Fries, 2005), and a neuronal state with stronger LRTC makes it easier to establish coherence, then transient functional connectivity established under strong LRTC would stabilize for longer durations, leading to a decrease in the number of percept reversals. This hypothesized role of LRTC in establishing long-range synchrony should be tested in a future study.

### LRTC and Power of Alpha Oscillations Are Regulated According to Task Goals

Irrmischer et al. (2017) showed that strong resting-state LRTC in the alpha band over the sensorimotor region were associated with fast reaction times in a subsequent sustained visual attention task. In our SFM task, nonetheless, how LRTC is associated with performance depends on the task requirements, with weaker alpha LRTC correlating with better performance when the goal is to increase the number of switches, and stronger alpha LRTC resulting in better performance when the goal is to decrease the number of switches. Leopold and Logothetis (1999) suggested that the spontaneous percept reversals occurring during multistable perception reflect an internal mechanism for exploring stimulus representations. Taken in this context, the proposal of Aston-Jones and Cohen (2005), that neuromodulation regulates task performance by promoting exploitation or exploration of alternatives, makes it a candidate mechanism for regulating neural dynamics in line with the SFM task goals.

How might neuromodulation alter LRTC? Computational modeling has shown that LRTC can emerge from a balance between excitatory and inhibitory connectivity in neuronal networks producing oscillations (Poil et al., 2012). Recent extensions to this model have shown that neuromodulation can dynamically regulate LRTC presumably by shifting the excitation/inhibition balance (Pfeffer et al., 2018). This was supported, in the same study, by pharmacological modulation showing that potentiation of the catecholaminergic system leads to increased LRTC both during eyes-open rest and during a structure-from-motion task. Surprisingly, Pfeffer et al. (2018) reported an increase in the number of perceptual switches when pharmacologically increasing LRTC of alpha oscillations in occipital and parietal regions, which is unexpected in view of the negative association that we report here for the within-subject relationship between LRTC and perceptual switches. It is likely that the pharmacological manipulation in Pfeffer et al. (2018) affects aspects of neuronal dynamics other than LRTC in a different manner from our study, resulting in the observed discrepancy. It is known that activity in the locus-coeruleus norepinephrine system is related to the level of arousal (Rajkowski et al., 1994; Aston-Jones et al., 2007). As such, administration of atomoxetine may result in more stringent changes in arousal, impinging on perception, than the dynamical self-regulation of neuronal activity present in our study. Also, atomoxetine may affect differentially the interaction between neuronal populations coding for competing percepts. Notably, while atomoxetine administration is not followed by changes in alpha power during the SFM task (Pfeffer et al., 2018), in our study, alpha power increases significantly between the Neutral and the Hold condition. Still, we expect that in the cortical state with LRTC elevated by means of atomoxetine, the same results as in our study should hold: subjects with higher LRTC should experience more stable percepts, and regulation of the number of switches due to task requirements should be accompanied by changes in LRTC.

### Discontinuity of Mind Not Predictive of Behavior in Bistable Perception

We found no evidence for our second hypothesis, which predicted that subjects with less Discontinuity of Mind during rest should experience less switches. This hypothesis was in part motivated by intuition and in part by preliminary evidence from our lab showing a negative association between LRTC in the alpha band and Discontinuity of Mind; however, this effect may not be sufficiently strong to show up in the present experiments. After all, we noted that the cortical state during ECR is radically different from that during the SFM tasks (Figures 6C,F).

### Outlook

Our study shows that individual differences in ongoing alpha power and LRTC can be regulated according to task goals, and are predictive of SFM task performance. While the impact of alpha power on perception has been the subject of extensive research, much less is known about how LRTC influences perception. In this respect, our data suggests that bistable visual perception is to some extent under voluntary control and influenced by LRTC of alpha oscillations. Thus, it seems that not only the level of noise but also the temporal structure of the noise in neural activity should be considered as an important aspect of neural dynamics, responsible for variation in perception and behavior.

## Author Contributions

FSI is involved in recording, processing, analysis of data and writing. A-EA is involved in data analysis and writing. MI is involved in conceptualizing, conducting, recording, processing and analysis of data. S-SP is involved in data analysis. HDM is involved in supervision and writing. KL-H is involved in conceptualizing, analyzing, supervision and writing.

## Conflict of Interest Statement

S-SP was employed by company NBT Analytics BV. The other authors declare that the research was conducted in the absence of any commercial or financial relationships that could be construed as a potential conflict of interest.

## Abbreviations

ARSQ: Amsterdam Resting-State Questionnaire
DFA: detrended fluctuation analysis
ECR: eyes-closed rest
EEG: electroencephalography
LRTC: long-range temporal correlations
SFM: structure from motion.
